# Coaxial Electrohydrodynamic Atomization for the Production of Drug-Loaded Micro/Nanoparticles

**DOI:** 10.3390/mi10020125

**Published:** 2019-02-14

**Authors:** Chuanpin Chen, Wenfang Liu, Ping Jiang, Tingting Hong

**Affiliations:** School of Pharmaceutical Sciences, Central South University, Changsha, Hunan 410013, China; ccpin2000@hotmail.com (C.C.); liuwenfang@csu.edu.cn (W.L.); pingj05@csu.edu.cn (P.J.)

**Keywords:** micro/nanoparticles, coaxial electrohydrodynamic atomization, drug delivery

## Abstract

Coaxial electrohydrodynamic atomization (CEHDA) presents a promising technology for preparing drug-loaded micro/nanoparticles with core-shell structures. Recently, CEHDA has attracted tremendous attention based on its specific advantages, including precise control over particle size and size distribution, reduced initial burst release and mild preparation conditions. Moreover, with different needles, CEHDA can produce a variety of drug-loaded micro/nanoparticles for drug delivery systems. In this review, we summarize recent advances in using double-layer structure, multilayer structure and multicomponent encapsulation strategies for developing micro/nanoparticles. The merits of applying multiplexed electrospray sources for high-throughput production are also highlighted.

## 1. Introduction

Much more attention has been paid to developing anticancer drugs in recent years as cancer is one of the most serious diseases threatening human health. However, the therapeutic effect of drugs is usually affected by their solubility, bioavailability and toxicity. Therefore, only one out of 5000–10,000 possible drugs is approved by the U.S. Food and Drug Administration (FDA) [1]. In order to improve the pharmacological effects of new drugs, a nano drug delivery system provides an efficient platform for development of the pharmaceutical industry. Efforts have been made to develop several traditional drug micro/nano-technology preparation methods, including emulsion crosslinking, ion crosslinking, compound crosslinking, emulsification-solvent evaporation, etc. In comparison with traditional strategies, the single-capillary electrostatic spraying method has been used to prepare drug-loaded particles with a higher drug entrapment rate and shortened time [2]. Electrostatic spraying can be seen as a "one step" method for obtaining drug-loaded micro/nanoparticles, which have a narrow size distribution range and better self-dispersibility. A simple preparation process and low operation costs are achieved using this technology [3]. In addition, this method has fewer restrictions on the applied materials for preparing the micro/nanoparticles, providing a potentially common technique for the development of nano drug delivery systems.

Although the single-capillary electrostatic spraying method exhibits specific advantages, there are still some limitations for preparing drug-loaded polymeric particles utilizing this strategy. During the process of spraying the drug-polymer mixture, the phenomenon of initial burst release is usually observed because of the surface/near-surface drug loading [4,5,6,7]. In the process of ejecting liquid onto the receiver, drugs are present on the surface and inside the particles when the solvent is completely volatilized. Drugs that stay on the surface of the carrier via physical adsorption and adhesion may easily cause drug release phenomenon [8]. Preparing drug-loaded particles with a core-shell structure is an appropriate way to introduce drugs directly into the core layer of the particles. Meanwhile, the shell polymer can protect the drugs in the nuclear layer to a certain extent. When these drug-loaded particles are intravenously injected into the human body, drugs can be released slowly from the nuclear layer with the continuous degradation of the shell material. Therefore, this strategy can effectively overcome sudden drug release behavior, solving the problem of ordinary electrostatic spray particles., Coaxial electrohydrodynamic atomization (CEHDA) provides a promising technology for achieving drug-loaded particles with a core-shell structure, and has attracted tremendous interest from researchers in recent years [9,10,11,12,13,14,15,16,17,18].

Wang et al. concluded that the CEHDA technique was effective for the fabrication of composite microparticles in 2015 [19]. We aimed to further summarize recent advances in the application of coaxial electrohydrodynamic atomization for producing drug-loaded micro/nanoparticles, focusing on double-layer structure, multilayer structure and multicomponent encapsulation strategies. Moreover, the advantages of employing multiplexed electrospray sources for high-throughput production are also discussed.

## 2. Concept of CEHDA

**Figure 1:**
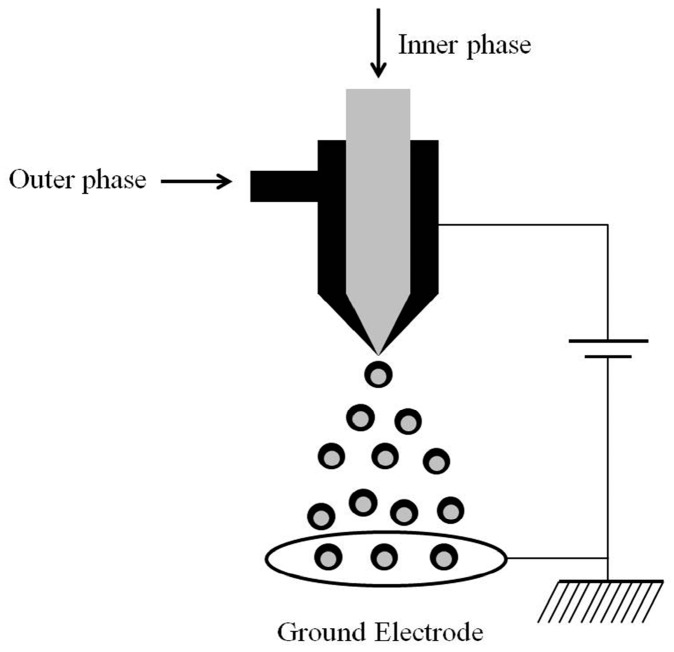
Typical experimental setup.

Coaxial electrospray, also called CEHDA, has been widely used in the preparation of drug-loaded biodegradable polymer particles and microbubbles for controlled and sustained drug release applications [20,21]. Figure 1 shows the typical experimental setup of CEHDA. A coaxial nozzle with multiple needles of different diameters is used to dispense different conducting liquids simultaneously by applying a high potential. An external electric field is utilized to adjust the formation process of droplets. During the operation progress, the electric field induces surface charging of the liquid at the tip of the nozzle, and the liquid is transformed into a conical shape, called a Taylor cone [22]. In addition, a grounded electrode is included in this device. Depending on the properties of the liquid, the liquid flow rate and the applied electric potential, different modes of CEHDA (e.g., dripping, cone-jet or multi-jets) can be developed. The cone-jet mode is one of the most popular CEHDA types for the production of uniform-sized particles. For drug-loaded particles, narrowly dispersed particles are able to provide precisely controlled drug release with minimum batch-to-batch variations. Furthermore, different nozzles can produce a variety of microparticles for the delivery of various drugs. Compared with monoaxial electrospraying, the CEHDA technique can achieve complete drug encapsulation, desirable control of release kinetics, and better drug stability. Moreover, it is easier to obtain monodispersity of particles using CEHDA instead of applying typical emulsion methods. In the drug delivery field, CEHDA exhibits tremendous advantages including precise control over the particle size and distribution with satisfactory repeatability, and flexibility in the types of drugs that can be encapsulated. The CEHDA technology presents promising potential in the fabrication of drug-loaded particles. However, there are challenges in preparing multi-layered particles. For instance, synthesizing core-shell particles using CEHDA with functional design in both core and shell phases is challenging. To prepare multidrug loaded microparticles, the maximum number of layers is affected by the interfacial tension and phase separation of the material solution in each layer.

**Figure 2:**
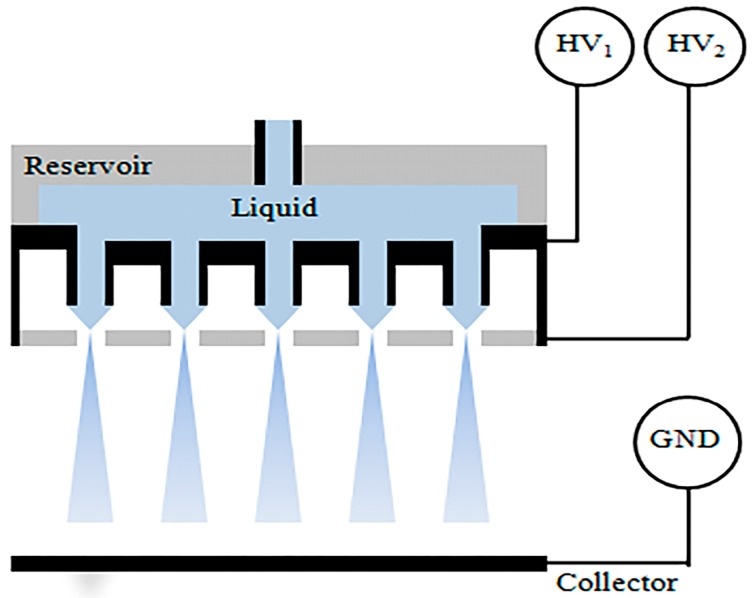
Multiplexed electrospray setup (Adapted from [24]).

At present, micro-scale CEHDA equipment with different needles has been fabricated to produce nanoparticle structures corresponding to various drug delivery requirements. However, standard coaxial electrospray sources cannot achieve high throughput since they have only one emitter. Low production efficiency is a shortcoming of electrospray equipment for industrial production. Therefore, expanding the production scale of CEHDA has become a popular research direction in recent years. The emitter is usually limited to a low flow rate because this allows the complete evaporation of polymer solvent for the core and/or shell preparation. In addition, the diameter of the particles increases with increasing flow rate. The parallel operation of the coaxial emitter array is a good way to increase the throughput of the coaxial ejection source without affecting particle size. Numerous studies have reported on developing multiple MEMS devices with uniaxial electrospray. A CEHDA scaling up study by Regele et al. showed that a four capillary array could increase the throughput by adding the fluid flux [23]. The results also indicated that the electric potential required for the formation of a stable Taylor cone increased as the capillary spacing decreased and vice versa. However, the four capillary nozzles prepared were still far from meeting the needs of large-scale production. Subsequently, Deng et al. developed a system consisting of multiple liquid dispensers of electrospray sources to increase production (Figure 2). The system was very compact and had a space density of up to 250 sources/cm^2^ [24,25].

Deng et al. further developed a well-controlled electrospray drying method to generate poly(lactic-co-glycolic acid) (PLGA) particles with different morphologies [26]. The results demonstrated that the order of polymer entanglement and coulomb fission in droplets could be controlled by optimizing the polymer molecular weight, concentration and solution flow rate, further adjusting the morphology of the resulting polymer particles. The expansion of synthetic polymer particles using multiple electrospray systems was favorable for practical applications. However, Bocanegra et al. found that in a multi-needle system, shielding phenomenon occurred near the surface of some conical menisci which could cause loss of the conical shape [27]. Therefore, the key issue for commercialize multiple electrospraying techniques is the design of a device that reduces the interference between adjacent needles which destroys the stable cone-jet on each needle. Parhizkar et al. proved that a circularly distributed needle array more easily achieved high particle size uniformity in comparison with a rectangular array while a lower voltage was required under the same operating conditions [28]. The scaled-up electrospray system has been applied in agriculture, sanitation, and other industrial applications [29,30,31,32,33,34,35].

**Figure 3:**
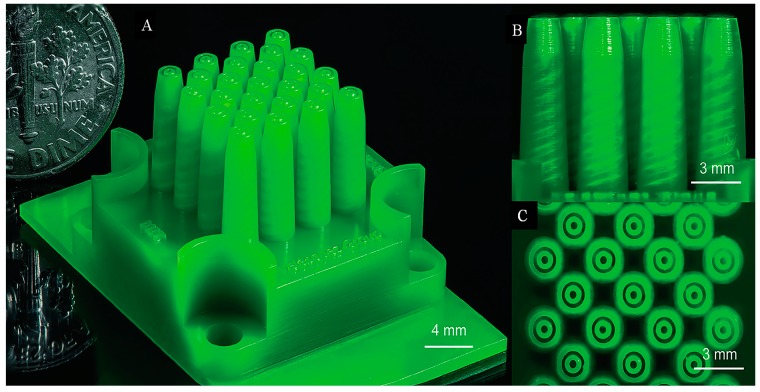
(**A**) Optical image of a 3D-printed planar array of 25 MEMS coaxial electrospray emitters in 1 cm^2^ of active area with a US dime coin for comparison. (**B**) Side view of the device, showing the tapered helical channels inside the tapered emitters. (**C** Top view of the device, showing the array of coaxial nozzles (Adapted from [36]).

Unlike uniaxial electrospraying, there have been few studies about microarray sources for coaxial electrospray. As we know, research about MEMS multiplexed coaxial electrospray was reported in 2016 [36]. Core-shell particle generators with up to 25 coaxial ejection emitters (25 emitters cm^−2^) were 3D-printed using stereolithography (Figure 3). 

The core/shell diameter and size distribution of the resulting compound particles could be flexibly adjusted online by controlling the flow rate supplied to the emitter. The throughput could achieve as high as 1,720,000 droplets per second. However, the microparticles prepared in this study had a minimum size of 17 μm and nanoscale particles could not be obtained. Moreover, the reported device was not resistant to a variety of solvents, including tetrahydrofuran, chloroform, and acetone. Though the suitability of such a device was limited as far as drug delivery applications, this report provided a new idea for future scale-up of the coaxial electrospray system.

### 2.1. Double-layer Structure Encapsulation

Double-layer structure encapsulation was reported for the first time in 2002 [37]. This is a microencapsulation technology based on electrohydrodynamic jetting of two immiscible liquids, which allows precise control over the geometry of the core-shell particles in a low size variation. A coaxial nozzle with two needles was arranged coaxially for preparing two-layer core-shell microparticles. Two immiscible liquids were injected at appropriate flow rates through two concentrically located needles. The outer needle was connected to an electrical potential of several kilovolts relative to a ground electrode to obtain a cone-jet mode. This technique was successfully applied to prepare monodisperse capsules with diameters varying between 10 and 0.15 micrometers. In addition, the composition of the core-shell particles could be changed by changing the contents of the injection, and the thickness and distribution of the layers could be optimized by adjusting the flow rate of the syringe pump. Although the monodispersity of the capsules prepared by Loscertales et al. did not reach the desired state, the work had a profound effect on subsequent studies. Later, a variety of micro/nano-particles were successfully developed by changing the core drugs and shell materials.

Xie et al. applied CEHDA technology to encapsulating biomacromolecules, avoiding the denaturation and aggregation effects of biological drugs when using conventional methods [38]. Bovine serum albumin and lysozyme, as model drugs, were encapsulated in polymer microparticles. The obtained particles were released in vitro for more than 30 days, and the released lysozyme activity was higher than 90%. The results were better than the related work reported in the previous study. Wu et al. succeeded in producing oligodeoxynucleotide (ODN) encapsulated lipoplex nanoparticles for gene delivery [39]. The particle size was reduced to 190 ± 39 nm, while the entrapment efficiency was increased to 90 ± 6%. Two years later, Bakhshi et al. reported a high-yield CEHDA one-step method for generating insulin-loaded polymeric nanoparticles, with a minimum particle size as low as 50 nm [40]. Factors affecting particle size were investigated. It was observed that larger droplets could be obtained with an increase in polymer concentration. The enhanced solvent volatilization was achieved by increasing the collection distance, further obtaining the minimum size. CEHDA technology was also used to encapsulate water-soluble first-line antiretroviral didanosine (ddI) in poly (epsilon-caprolactone) (PCL) particles, and stabilized it in the gastric medium [41]. Compared with other reports, its load capacity was relatively high (about 12% w/w), and the encapsulation efficiency was also up to about 100%. This study led to a significant increase in the oral bioavailability of almost four times and a 2-fold extension of the half-life with compared to a ddI aqueous solution. In the same year, Ang II was encapsulated into tristearin core-shell nanoparticles (NPs) (average size 100–300 nm) via a coaxial electrospray technique, and encapsulation efficiency of 92 ± 1.8% was obtained [42]. The MTT toxicity test effectively determined the acceptable load concentration of the loaded or unloaded packaged nanoparticles, which did not produce acute toxicity or morphological effects in vitro. Gallovic et al. proved that it was possible to increase the survival rate of inhaled *Bacillus anthracis* by using the acetyl glucan microparticle vaccine prepared by coaxial electrospray [43]. The antigenicity of the vaccine was improved during the formulation and administration process. Numerous reports indicated that CEHDA could be a reproducible and cost-effective technique for encapsulating biological macromolecules and subunit vaccines [44,45,46,47].

In addition, CEHDA technology also exhibited its outstanding performance for preparing chemical drug-loaded particles [48]. Budesonide and water-soluble polyphenols were encapsulated in monodisperse and uniformly-sized poly(lactic-co-glycolic acid) (PLGA) nanoparticles, respectively. The obtained particle sizes ranged from 165 nm to 1.2 μm. The results indicated that the application of CEHDA was not limited to drug solubility. Furthermore, the mechanism of releasing the nanoparticles was studied. It was observed that the drug release rate decreased as the nanoparticle size increased. The initial drug release behavior of these sub-micron particles prepared using the dual-capillary electrospray method was mainly due to water permeation and drug diffusion, rather than PLGA degradation. In comparison with conventional strategies, the electrospray method exhibited specific advantages for developing drug-loaded particles. Since the core-shell structure of the particles could prevent the drugs from being absorbed on the surface of the particles and/or encapsulated near the surface of the particles, the drugs had minimal or no initial burst release. Complete drug release was obtained due to no polyvinyl alcohol (PVA) being involved in the electrospray process. Researchers also found the diameter of drug-loaded polymer particles could be adjusted by controlling the concentration and electrical conductivity of PLGA solutions. New methods were investigated to tune the size of drug-loaded nanoparticles for meeting different requirements. 

**Figure 4:**
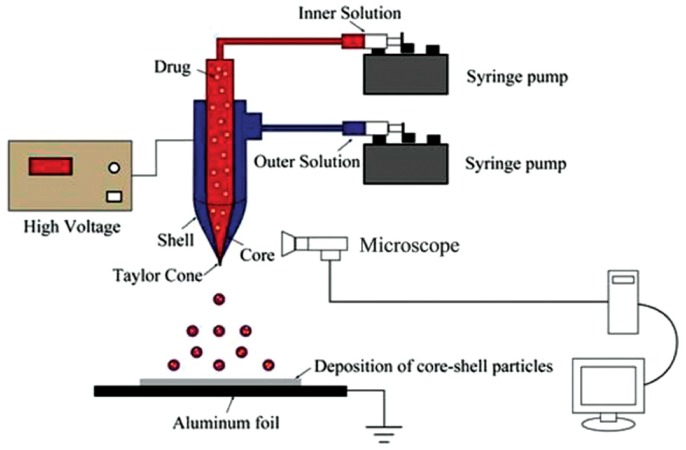
Experimental setup for preparing core-shell nanoparticles (Adapted from [49]).

In order to achieve targeted chemotherapy for pancreatic cancer, Xu et al. prepared core-shell nanoparticles containing gemcitabine via CEHDA technology [49,50]. Figure 4 illustrates the working mechanism of the electrosprayer for preparing core-shell nanoparticles. By optimizing the electrospray parameters, the diameter of the prepared folate conjugated core-shell nanoparticles was in the range of 200 to 300 nm, and the drug loading and encapsulation efficiency were about 3.91 ± 0.12% and 85.37 ± 4.9%, respectively. Cytotoxicity tests showed that the obtained particles had a significant effect on the cytotoxicity of BXPC3 cells. It demonstrated that folate-conjugated core-shell nanoparticles were effective in targeting a pancreatic tumor. In addition, doxorubicin could also be encapsulated in the polymer shell with CEHDA. In particular, the researchers applied PVA solution as the carrier stream, and the middle and inner layers were poly(L-lactic acid) PLLA solution and PLGA solution, respectively. The core-shell microparticles were removed by removing residual PVA. Unfortunately, the obtained particle size was as large as 66–75 microns. Therefore, Cao et al. further optimized the flow rate, solution concentration, and other conditions on the basis of the former work, and the prepared paclitaxel nanoparticles were as low as 106 ± 5 nm [51]. This study proved that nanoparticles had good dispersion stability and low cytotoxicity in water, which could improve paclitaxel water solubility and decrease side effects. In the next few years, drugs including acyclovir, estradiol, paclitaxel, adriamycin, artesunate, rifampicin, and metronidazole were encapsulated in porous nanoparticles [52,53,54,55,56,57,58]. This suggested that CEHDA technology could be applied to develop various drug-loaded nanoparticles.

### 2.2. Multilayer Structure Encapsulation

The emergence of CEHDA has shown prominent prospects for the production of core-shell granules at microscopic and nanoscale scales. With the increased demand for developing a multilayer structure, further efforts were made to enhance current technology for various applications. This work proved that CEHDA could produce complex structures in nanometer and micrometer sizes [59]. A novel device was fabricated by using three coaxial aligned needles. According to the structure of the desired nanoparticles, the corresponding solution was injected into different needles, and the electric field was connected to the electrostatic spraying. The double-layer bubbles, porous encapsulation threads, and three-layer nanocapsules were successfully prepared by changing the experimental conditions including the concentration of glycerol, olive oil, polyethylene oxide (PEO) and other materials.

**Figure 5:**
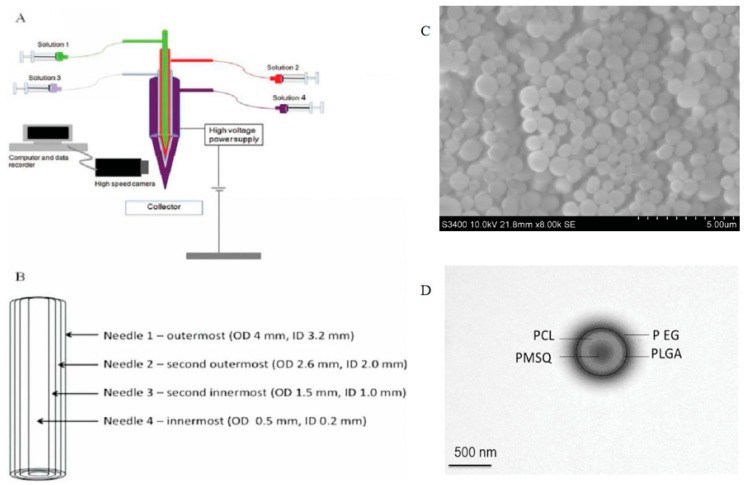
Schematic illustration of (**A**) the experimental set-up of the EHD process using a four-needle device for forming four-layer structures with a stable jet (inset); (**B**) the coaxial needle arrangement with labeled dimensions, where ID and OD are internal and outer diameters, respectively; (**C**) SEM image of four-layered particles at low magnification; (**D**) bright-field TEM image of a particle showing four distinct layers (Adapted from [64]).

Three biocompatible polymer solutions of PLGA, polycaprolactone (PCL) and polymethyl silsesquioxane (PMSQ) were used to prepare monodisperse, spherical submicron particles [60,61,62,63]. After increasing the working distance from 50 to 350 mm, spherical particles with an average particle size of 320 (± 80 nm) to 220 (± 8 nm) were obtained. It was demonstrated that the size distribution became narrower as the working distance increased. In addition, the particles were non-cytotoxic, indicating their potential for medical applications. In 2014, this group further produced a novel four-needle coaxial electrohydrodynamic (EHD) device (Figure 5). A layer of polyethylene glycol (PEG) shell was added in the outer layer of nanoparticles, and nanoparticles with better stability and increased average size (620 ± 150 nm) were achieved [64]; particles with a four-layer structure were also obtained. Recently, three-layered nanoparticles with an ideal size for drug delivery were prepared with a four-needle coaxial electrohydrodynamic device [65]. In this study, cisplatin and fluorescently labeled siRNA were chosen as the model therapeutic agents. Researchers produced about 130 nm of nanoparticles with three distinct layers which contained an outer protective PLGA layer, an intermediate layer of siRNA, and an inner layer of cisplatin. This three-layer nanoparticle provided a desirable environment for the joint management of low molecular weight chemotherapeutic agents and the reduction of nucleic acid resistance. It proved that it was possible to produce separated multilayered nanoparticles that could meet different structural and environmental requirements for larger scale production and drug delivery.

### 2.3. Multicomponent Encapsulation

**Figure 6:**
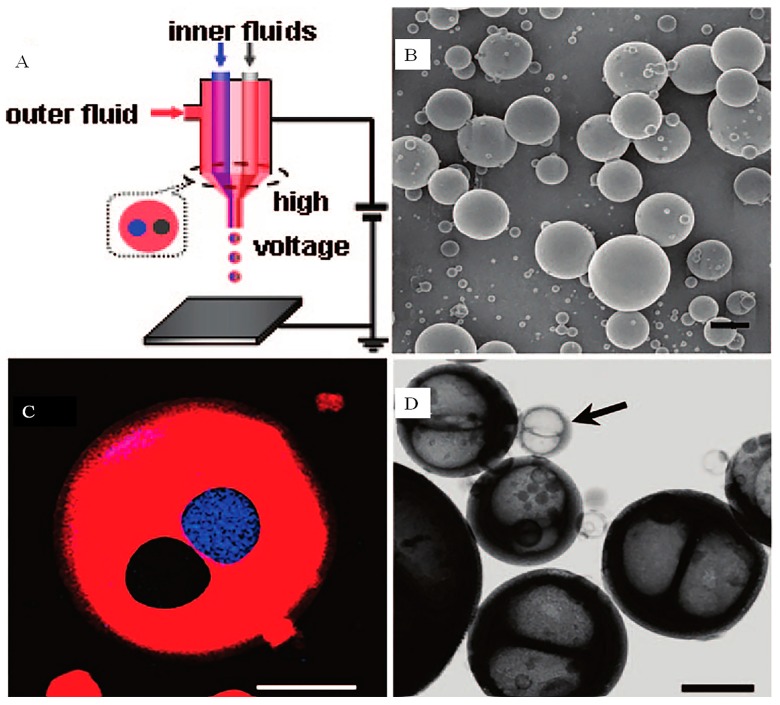
(**A**) Illustration of bicomponent microcapsule fabrication system. Two core liquids (blue and gray) were pumped out from two inner metal capillaries, respectively, and shell liquid (red) flowed through gaps between inner capillaries and the outer needle. (**B**) SEM image of titania composite capsules, which ranged from submicrometer to several micrometers. Scale bar: 2 µm. (**C**) LSCM overlay image of titania composite capsules. The two core contents have been inhibited into individual compartments without contact. Scale bar: 10 µm. (**D**) TEM image of “θ” structured titania bicompartment microcapsules after organics have been removed by calcination. The smallest capsule is only hundreds of nanometers as indicated by the arrow. Scale bar: 1 µm (Adapted from [76]).

A variety of effective chemical or physical encapsulation methods, including microfluidics, self-assembly, emulsion, flow focusing technologies and promising electrical coaxial jet technology have been developed for drug delivery [66,67,68,69,70,71,72,73,74,75]. However, most of these encapsulation methods use two types of materials (core and shell), that is, only one content can be encapsulated at a time. In order to overcome this drawback, some researchers have been working on the preparation of microcapsules that could encapsulate a wide variety of contents at one time. Chen et al. developed a composite fluid electrospray device that allowed multiple components to be encapsulated in one-step in a single microcapsule [76]. This device was fabricated with a layered composite nozzle which was assembled by separately embedding two metal capillaries into a blunt metal needle (Figure 6a). The capsules with diameters above 10 μm were obtained and the internal structure of the capsules was detected with a microscope. As shown in Figure 6, the transmission electron microscopy (TEM) images also confirmed that the new dual-chamber structure, just like the Greek character ‘θ’, was obtained with continuous depression embedding.

A composite nozzle was further assembled by using three internal capillaries and three different core fluids (red, blue and yellow dyed glycerol), respectively. In this work, three different components could be encapsulated in the microcapsules at one time [77]. Si et al. prepared a multi-core microcapsule of about 100 microns with a similar CEHDA device, using stained paraffin oil and alginic acid as model materials. The device could be applied to packaging cells, therapeutic agents, and also drugs [78]. 

**Figure 7:**
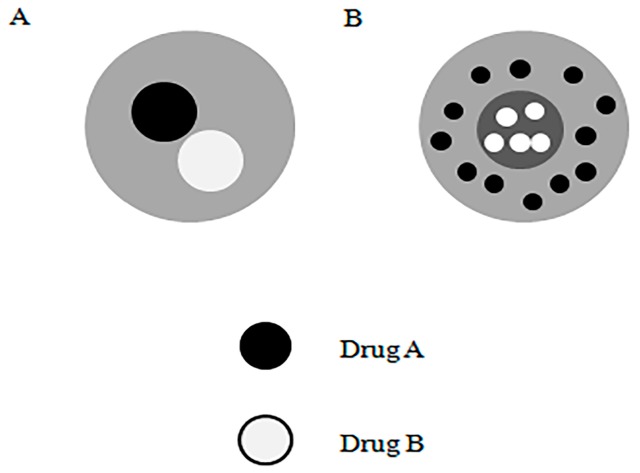
A brief comparison of the structure of (**A**) multi-compartment microparticles, and (**B**) multilayered microparticles loaded with multiple drugs at the same time (Adapted from [84]).

Another common multi-component encapsulation method could be used in combination therapy to minimize cytotoxicity as well as to maximize cell resistance [79]. Recent in vitro cellular tests and in vivo animal experiments can offer important data to optimize particles for the desirable therapeutic efficacy [80,81]. Clinical reports indicated that paclitaxel and suramin had a cumulative effect on the treatment of solid tumors [82,83]. However, high initial concentrations and/or rapid release of suramin might cause serious toxicity to surrounding normal cells. Therefore, similar to the case of paclitaxel, high initial concentrations of suramin were not recommended for rapid release. In order to tackle these challenges, microspheres releasing multiple drugs in a controlled manner were highly demanded. Paclitaxel and suramin were encapsulated by core-shell nanoparticles using multi-axis electrospray [84,85]. This method allowed the encapsulation of two drugs with different hydrophilic properties in a single step. The structure of this capsule was different from the multi-compartment capsule mentioned earlier, which mixed doxorubicin and paclitaxel in the innermost layer and the second layer of shell material solution, and the outermost layer was PLGA shell for reducing the initial rupture release (Figure 7). 

**Figure 8:**
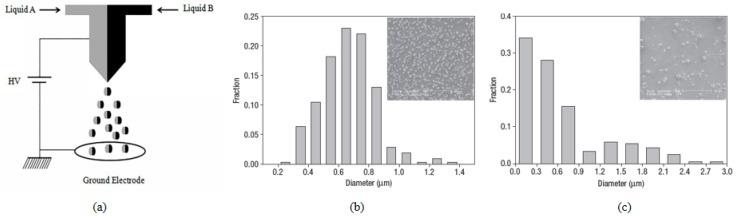
(**a**) Schematic image of the electrohydrodynamic co-jetting process yielding bicompartmental spherical, discoid, and rod-shaped microparticles; (**b**) Size distributions of polyethylene oxide (PEO); and (**c**), polyacrylic acid (PAA) biphasic particles determined from the SEM images (Adapted from [92]).

However, the size of the produced capsules was mostly in the tens of microns, and such a large particle size might hinder the application of these capsules. Similarly, for tumor chemotherapy, the results suggested that the combination of drugs such as paclitaxel and doxorubicin could increase the maximum tolerated dose and tumor regression rate [86,87,88,89]. Therefore, Kim et al. used a triaxial capillary ejection device to produce biodegradable multi-shell capsules for constructing drug delivery systems [90]. Capsules formed by triaxial electrospray systems could release a variety of drugs in a single step, in which the release rate of each drug is independently controlled by varying the capsule diameter and the shell thickness. In addition, the initial outbreak was significantly reduced, paclitaxel and doxorubicin were released with a stable zero-order distribution. Due to the flexible control of multiple drugs and the different release rates, the multi-shell capsules showed great potential as a drug delivery system. This technique facilitated the reduction of drug initial outbreak release, and drug dose quantity and frequency. Later, naproxen and rhodamine B (RH.B) were encapsulated in nanoparticle core and shell layers to achieve multiple drug delivery systems with controlled release [91]. The success of preparing particles in nanosize provided a satisfactory carrier for further applications. Besides, Lahann et al. developed biphasic Janus particles and triphasic nano colloids with nanoscale anisotropy by using a modified nozzle with side-by-side geometry (Figure 8) [92,93,94,95,96,97,98,99,100,101]. This method could be extended to the manufacture of multi-compartmental particles including side by side, pie-shaped, asymmetric, striped and rosette [102].

## 3. Conclusions

In comparison with other available chemical and physical methods, CEHDA technology exhibits tremendous advantages for preparing micro/nanoparticles in the area of drug delivery. Specific merits including: (1) Precise control over the particle size and distribution with high reproducibility; (2) encapsulation of therapeutic agents in microparticle core with a polymer shell, reducing the high initial burst release; (3) optimization of drug release rate and drug targeted therapy by selecting appropriate materials and controlling the thickness of the shell; (4) utilization of mild preparation conditions without using emulsifiers. In general, these advantages demonstrate the promising potential of CEHDA technology for producing drug loaded micro/nanoparticles with high reproducibility and scalability. The obtained particles with a core-shell structure facilitate sequential release of anti-angiogenic agents and anticancer drugs, which may be more effective in treating tumors. Side effects of the drugs can be eliminated by targeting therapy using modified particles. In addition, CEHDA provides a desirable platform for using smart materials, including pH-responsive materials and temperature-sensitive materials for drug delivery. Future studies should focus on developing multichannel composite injection source and using CEHDA to develop multifunctional particles for combination therapy, diagnosis, targeted drug delivery and treatment response monitoring.

## References

[1] Kola I., Landis J. (2004). Can the pharmaceutical industry reduce attrition rates?. Nat. Rev. Drug Discov..

[2] Nagpal K., Singh S.K., Mishra D.N. (2010). Chitosan nanoparticles: A promising system in novel drug delivery. Chem. Pharm. Bull..

[3] Chakraborty S., Liao I.C., Adler A., Leong K.W. (2009). Electrohydrodynamics: A facile technique to fabricate drug delivery systems. Adv. Drug Deliv. Rev..

[4] Wu Y., MacKay J.A., McDaniel J.R., Chilkoti A., Clark R.L. (2009). Fabrication of elastin-like polypeptide nanoparticles for drug delivery by electrospraying. Biomacromolecules.

[5] Deng W., Waits C.M., Morgan B., Gomez A. (2009). Compact multiplexing of monodisperse electrosprays. J. Aerosol. Sci..

[6] Bhatnagar P. (2007). Multiplexed electrospray deposition for protein microarray with micromachined silicon device. Appl. Phys. Lett..

[7] Xie J., Wang C. (2007). Encapsulation of proteins in biodegradable polymeric microparticles using electrospray in the Taylor Cone-Jet mode. Biotechnol. Bioeng..

[8] Park J.H., Saravanakumar G., Kim K., Kwon I.C. (2010). Targeted delivery of low molecular drugs using chitosan and its derivatives. Adv. Drug Deliver. Rev..

[9] Liu W., Chen X.D., Selomulya C. (2015). On the spray drying of uniform functional microparticles. Particuology.

[10] Mehta P., Haj-Ahmad R., Rasekh M., Arshad M.S., Smith A., van der Merwe S.M., Li X., Chang M., Ahmad Z. (2017). Pharmaceutical and biomaterial engineering via electrohydrodynamic atomization technologies. Drug Discov. Today.

[11] Liu Z.P., Zhang Y.Y., Yu D.G., Wu D., Li H.L. (2018). Fabrication of sustained-release zein nanoparticles via modified coaxial electrospraying. Chem. Eng. J..

[12] Wan F., Yang M. (2016). Design of PLGA-based depot delivery systems for biopharmaceuticals prepared by spray drying. Int. J. Pharmaceut..

[13] Xie J., Jiang J., Davoodi P., Srinivasan M.P., Wang C. (2015). Electrohydrodynamic atomization: A two-decade effort to produce and process micro-/nanoparticulate materials. Chem. Eng. Sci..

[14] Sosnik A. (2014). Production of drug-loaded polymeric nanoparticles by electrospraying technology. J Biomed. Nanotechnol..

[15] Sridhar R., Ramakrishna S. (2013). Electrosprayed nanoparticles for drug delivery and pharmaceutical applications. Biomatter.

[16] Sengupta S., Eavarone D., Capila I., Zhao G.L., Watson N., Kiziltepe T., Sasisekharan R. (2005). Temporal targeting of tumour cells and neovasculature with a nanoscale delivery system. Nature.

[17] Cui Y., Xu Q., Chow P.K., Wang D., Wang C. (2013). Transferrin-conjugated magnetic silica PLGA nanoparticles loaded with doxorubicin and paclitaxel for brain glioma treatment. Biomaterials.

[18] Eltayeb M., Stride E., Edirisinghe M., Harker A. (2016). Electrosprayed nanoparticle delivery system for controlled release. Mat. Sci. Eng. C-Mater.

[19] Davoodi P., Feng F., Xu Q., Yan W., Tong Y.W., Srinivasan M.P., Sharma V.K., Wang C. (2015). Coaxial electrohydrodynamic atomization: Microparticles for drug delivery applications. J. Control Release.

[20] Rasekh M., Ahmad Z., Cross R., Hernández-Gil J., Wilton-Ely J.D.E.T., Miller P.W. (2017). Facile preparation of drug-loaded tristearin encapsulated superparamagnetic iron oxide nanoparticles using coaxial electrospray processing. Mol. Pharmaceutics.

[21] Yan W.C., Ong X.J., Pun K.T., Tan D.Y., Sharma V.K., Tong Y.W., Wang C.H. (2017). Preparation of tPA-loaded microbubbles as potential theranostic agents: A novel one-step method via coaxial electrohydrodynamic atomization technique. Chem. Eng. J..

[22] Raut J.S., Akella S., Singh A.K., Naik V.M. (2009). Catastrophic drop breakup in electric field. Langmuir.

[23] Regele J.D., Papac M.J., Rickard M., Dunn-Rankin D. (2002). Effects of capillary spacing on EHD spraying from an array of cone jets. J. Aerosol Sci..

[24] Deng W.W., Klemic J.F., Li X.H., Reed M.A., Gomez A. (2006). Increase of electrospray throughput using multiplexed microfabricated sources for the scalable generation of monodisperse droplets. J. Aerosol. Sci..

[25] Deng W., Gomez A. (2007). Influence of space charge on the scale-up of multiplexed electrosprays. J. Aerosol. Sci..

[26] Almeria B., Deng W., Fahmy T.M., Gomez A. (2010). Controlling the morphology of electrospray-generated PLGA microparticles for drug delivery. J. Colloid Interf. Sci..

[27] Bocanegra R., Galan D., Marquez M., Loscertales I.G., Barrero A. (2005). Multiple electrosprays emitted from an array of holes. J. Aerosol. Sci..

[28] Parhizkar M., Reardon P.J.T., Knowles J.C., Browning R.J., Stride E., Pedley R.B., Grego T., Edirisinghe M. (2017). Performance of novel high throughput multi electrospray systems for forming of polymeric micro/nanoparticles. Mater. Design.

[29] Almeria B., Fahmy T.M., Gomez A. (2011). A multiplexed electrospray process for single-step synthesis of stabilized polymer particles for drug delivery. J. Control Release.

[30] Lhernould M.S., Lambert P. (2011). Compact polymer multi-nozzles electrospray device with integrated microfluidic feeding system. J. Electrostat..

[31] Yang W., Lojewski B., Wei Y., Deng W. (2012). Interactions and deposition patterns of multiplexed electrosprays. J. Aerosol. Sci..

[32] Lenguito G., Gomez A. (2013). Development of a multiplexed electrospray micro-thruster with post-acceleration and beam containment. J. Appl. Phys..

[33] Hill F.A., Heubel E.V., de Leon P.P., Velasquez-Garcia L.F. (2014). High-throughput ionic liquid ion sources using arrays of microfabricated electrospray emitters with integrated extractor grid and carbon nanotube flow control structures. J. Microelectromech. S.

[34] Dandavino S., Ataman C., Ryan C.N., Chakraborty S., Courtney D., Stark J.P.W., Shea H. (2014). Microfabricated electrospray emitter arrays with integrated extractor and accelerator electrodes for the propulsion of small spacecraft. J. Micromech. Microeng..

[35] Lojewski B., Yang W., Duan H., Xu C., Deng W. (2013). Design, Fabrication, and Characterization of Linear Multiplexed Electrospray Atomizers Micro- Machined from Metal and Polymers. Aerosol. Sci. Tech..

[36] Olvera-Trejo D., Velasquez-Garcia L.F. (2016). Additively manufactured MEMS multiplexed coaxial electrospray sources for high-throughput, uniform generation of core-shell microparticles. Lab Chip.

[37] Loscertales I.G., Barrero A., Guerrero I., Cortijo R., Marquez M., Ganan-Calvo A.M. (2002). Micro/nano encapsulation via electrified coaxial liquid jets. Science.

[38] Xie J., Ng W.J., Lee L.Y., Wang C. (2008). Encapsulation of protein drugs in biodegradable microparticles by co-axial electrospray. J. Colloid Interf. Sci..

[39] Wu Y., Yu B., Jackson A., Zha W., Lee L.J., Wyslouzil B.E. (2009). Coaxial electrohydrodynamic spraying: A novel one-step technique to prepare oligodeoxynucleotide encapsulated lipoplex nanoparticles. Mol. Pharmaceut..

[40] Bakhshi R., Ahmad Z., Soric M., Stride E., Edirisinghe M. (2011). Nanoparticle delivery systems formed using electrically sprayed co-flowing excipients and active agent. J. Biomed. Nanotechnol..

[41] Seremeta K.P., Hoecht C., Taira C., Cortez Tornello P.R., Abraham G.A., Sosnik A. (2015). Didanosine-loaded poly(epsilon-caprolactone) microparticles by a coaxial electrohydrodynamic atomization (CEHDA) technique. J. Mater. Chem. B.

[42] Rasekh M., Young C., Roldo M., Lancien F., Le Mevel J., Hafizi S., Ahmad Z., Barbu E., Gorecki D. (2015). Hollow-layered nanoparticles for therapeutic delivery of peptide prepared using electrospraying. J. Mater. Sci-Mater M.

[43] Gallovic M.D., Schully K.L., Bell M.G., Elberson M.A., Palmer J.R., Darko C.A., Bachelder E.M., Wyslouzil B.E., Keane-Myers A.M., Ainslie K.M. (2016). Acetalated dextran microparticulate vaccine formulated via Coaxial Electrospray preserves toxin neutralization and enhances murine survival following inhalational Bacillus anthracis exposure. Adv. Healthc. Mater..

[44] Zamani M., Prabhakaran M.P., Thian E.S., Ramakrishna S. (2014). Protein encapsulated core-shell structured particles prepared by coaxial electrospraying: Investigation on material and processing variables. Int. J. Pharmaceut..

[45] Laelorspoen N., Wongsasulak S., Yoovidhya T., Devahastin S. (2014). Microencapsulation of Lactobacillus acidophilus in zein-alginate core-shell microcapsules via electrospraying. J. Funct. Foods.

[46] Sharma B., Takamura Y., Shimoda T., Biyani M. (2016). A bulk sub-femtoliter in vitro compartmentalization system using super-fine electrosprays. Sci. Rep..

[47] Xia Y., Pack D.W. (2014). Pulsatile protein release from monodisperse liquid-core microcapsules of controllable shell thickness. Pharm. Res-Dordr..

[48] Lee Y., Mei F., Bai M., Zhao S., Chen D. (2010). Release profile characteristics of biodegradable-polymer-coated drug particles fabricated by dual-capillary electrospray. J. Control Release.

[49] Xu S., Xu Q., Zhou J., Wang J., Zhang N., Zhang L. (2013). Preparation and characterization of folate-chitosan-gemcitabine core-shell nanoparticles for potential tumor-targeted drug delivery. J. Nanosci. Nanotechno..

[50] Xu Q., Chin S.E., Wang C., Pack D.W. (2013). Mechanism of drug release from double-walled PDLLA (PLGA) microspheres. Biomaterials.

[51] Cao L., Luo J., Tu K., Wang L., Jiang H. (2014). Generation of nano-sized core-shell particles using a coaxial tri-capillary electrospray-template removal method. Colloid Surface B.

[52] Liu Z., Cui L., Yu D., Zhao Z., Chen L. (2014). Electrosprayed core-shell solid dispersions of acyclovir fabricated using an epoxy-coated concentric spray head. Int. J. Nanomed..

[53] Enayati M., Ahmad Z., Stride E., Edirisinghe M. (2010). One-step electrohydrodynamic production of drug-loaded micro- and nanoparticles. J. R. Soc. Interface.

[54] Jing Y., Zhu Y., Yang X., Shen J., Li C. (2011). Ultrasound-triggered smart drug release from multifunctional core-shell capsules one-step fabricated by coaxial electrospray method. Langmuir.

[55] Xu Q., Qin H., Yin Z., Hua J., Pack D.W., Wang C. (2013). Coaxial electrohydrodynamic atomization process for production of polymeric composite microspheres. Chem. Eng. Sci..

[56] Hoang N.H., Laidmae I., Kogermann K., Lust A., Meos A., Chien N.N., Heinamaki J. (2017). Development of electrosprayed artesunate-loaded core-shell nanoparticles. Drug Dev. Ind. Pharm..

[57] Ghayempour S., Mortazavi S.M. (2013). Fabrication of micro–nanocapsules by a new electrospraying method using coaxial jets and examination of effective parameters on their production. J. Electrostat..

[58] Hao S., Wang B., Wang Y. (2015). Porous hydrophilic core/hydrophobic shell nanoparticles for particle size and drug release control. Mat. Sci. Eng C-Mater..

[59] Ahmad Z., Zhang H.B., Farook U., Edirisinghe M., Stride E., Colombo P. (2008). Generation of multilayered structures for biomedical applications using a novel tri-needle coaxial device and electrohydrodynamic flow. J. R. Soc. Interface.

[60] Makadia H.K., Siegel S.J. (2011). Poly lactic-co-glycolic acid (PLGA) as biodegradable controlled drug delivery carrier. Polymers-Basel.

[61] Quintanar-Guerrero D., Allemann E., Fessi H., Doelker E. (1998). Preparation techniques and mechanisms of formation of biodegradable nanoparticles from preformed polymers. Drug Dev. Ind. Pharm..

[62] Xiang H., Zhang L., Wang Z., Yu X., Long Y., Zhang X., Zhao N., Xu J. (2011). Multifunctional polymethylsilsesquioxane (PMSQ) surfaces prepared by electrospinning at the sol-gel transition: Superhydrophobicity, excellent solvent resistance, thermal stability and enhanced sound absorption property. J. Colloid. Interf. Sci..

[63] Labbaf S., Deb S., Cama G., Stride E., Edirisinghe M. (2013). Preparation of multicompartment sub-micron particles using a triple-needle electrohydrodynamic device. J. Colloid Interf. Sci..

[64] Labbaf S., Ghanbar H., Stride E., Edirisinghe M. (2014). Preparation of multilayered polymeric structures using a novel four-needle coaxial electrohydrodynamic device. Macromol. Rapid Comm..

[65] Pina M.F., Lau W., Scherer K., Parhizkar M., Edirisinghe M., Craig D. (2017). The generation of compartmentalized nanoparticles containing siRNA and cisplatin using a multi-needle electrohydrodynamic strategy. Nanoscale.

[66] Okushima S., Nisisako T., Torii T., Higuchi T. (2004). Controlled production of monodisperse double emulsions by two-step droplet breakup in microfluidic devices. Langmuir.

[67] Peyratout C.S., Dahne L. (2004). Tailor-made polyelectrolyte microcapsules: From multilayers to smart containers. Angew Chem. Int. Edit.

[68] Gref R., Minamitake Y., Peracchia M.T., Trubetskoy V., Torchilin V., Langer R. (1994). Biodegradable long-circulating polymeric nanospheres. Science.

[69] Utada A.S., Lorenceau E., Link D.R., Kaplan P.D., Stone H.A., Weitz D.A. (2005). Monodisperse double emulsions generated from a microcapillary device. Science.

[70] Larsen G., Velarde-Ortiz R., Minchow K., Barrero A., Loscertales I.G. (2003). A method for making inorganic and hybrid (organic/inorganic) fibers and vesicles with diameters in the submicrometer and micrometer range via sol-gel chemistry and electrically forced liquid jets. J. Am. Chem. Soc..

[71] Marin A.G., Loscertales I.G., Marquez M., Barrero A. (2007). Simple and double emulsions via coaxial jet electrosprays. Phys. Rev. Lett..

[72] Dror Y., Salalha W., Avrahami R., Zussman E., Yarin A.L., Dersch R., Greiner A., Wendorff J.H. (2007). One-step production of polymeric microtubes by co-electrospinning. Small.

[73] Bazilevsky A.V., Yarin A.L., Megaridis C.M. (2007). Co-electrospinning of core-shell fibers using a single-nozzle technique. Langmuir.

[74] Li X.H., Shao C.L., Liu Y.C. (2007). A simple method for controllable preparation of polymer nanotubes via a single capillary electrospinning. Langmuir.

[75] Zhao Y., Cao X., Jiang L. (2007). Bio-mimic multichannel microtubes by a facile method. J. Am. Chem. Soc..

[76] Chen H., Zhao Y., Song Y., Jiang L. (2008). One-step multicomponent encapsulation by compound-fluidic electrospray. J. Am. Chem. Soc..

[77] Chen H., Zhao Y., Jiang L. (2009). Compound-fluidic electrospray: An efficient method for the fabrication of microcapsules with multicompartment structure. Chin. Sci. Bull..

[78] Si T., Yin C., Gao P., Li G., Ding H., He X., Xie B., Xu R.X. (2016). Steady cone-jet mode in compound-fluidic electro-flow focusing for fabricating multicompartment microcapsules. Appl. Phys. Lett..

[79] Ahmed F., Pakunlu R.I., Brannan A., Bates F., Minko T., Discher D.E. (2006). Biodegradable polymersomes loaded with both paclitaxel and doxorubicin permeate and shrink tumors, inducing apoptosis in proportion to accumulated drug. J. Control Release.

[80] Chang P.C., Dovban A.S., Lim L.P., Chong L.Y., Kuo M.Y., Wang C.H. (2013). Dual delivery of PDGF and simvastatin to accelerate periodontal regeneration in vivo. Biomaterials.

[81] Chang P.C., Chong L.Y., Dovban A.S., Lim L.P., Lim J.C., Kuo M.Y., Wang C.H. (2014). Sequential platelet-derived growth factor-simvastatin release promotes dentoalveolar regeneration. Tissue Eng. A.

[82] Kruger E.A., Figg W.D. (2001). Protein binding alters the activity of suramin, carboxyamidotriazole, and UCN-01 in an ex vivo rat aortic ring angiogenesis assay. Clin. Cancer Res..

[83] Song S.H., Yu B., Wei Y., Wientjes M.G., An J. (2004). Low-dose suramin enhanced paclitaxel activity in chemotherapy-naive and paclitaxel-pretreated human breast xenograft tumors. Clin. Cancer Res..

[84] Nie H., Fu Y., Wang C. (2010). Paclitaxel and suramin-loaded core/shell microspheres in the treatment of brain tumors. Biomaterials.

[85] Nie H., Dong Z., Arifin D.Y., Hu Y., Wang C. (2010). Core/shell microspheres via coaxial electrohydrodynamic atomization for sequential and parallel release of drugs. J. Biomed. Mater. Res..

[86] Briasoulis E., Karavasilis V., Tzamakou E., Rammou D., Soulti K., Piperidou C., Pavlidis N. (2004). Interaction pharmacokinetics of pegylated liposomal doxorubicin (Caelyx) on coadministration with paclitaxel or docetaxel. Cancer Chemoth. Pharm..

[87] Gehl J., Boesgaard M., Paaske T., Vittrup Jensen B., Dombernowsky P. (1996). Combined doxorubicin and paclitaxel in advanced breast cancer: effective and cardiotoxic. Ann. Oncol. Off. J. Eur. Soc. Med. Oncol..

[88] Gustafson D.L., Merz A.L., Long M.E. (2005). Pharmacokinetics of combined doxorubicin and paclitaxel in mice. Cancer Lett..

[89] Mavroudis D., Kouroussis C., Kakolyris S., Agelaki S., Kalbakis K., Androulakis N., Souglakos J., Samonis G., Georgoulias V. (2002). Phase I study of paclitaxel (taxol) and pegylated liposomal doxorubicin (Caelyx) administered every 2 weeks in patients with advanced solid tumors. Oncol.-Basel.

[90] Kim W., Kim S.S. (2011). Synthesis of biodegradable triple-layered capsules using a triaxial electrospray method. Polymer.

[91] Wang Y., Zhang Y., Wang B., Cao Y., Yu Q., Yin T. (2013). Fabrication of core-shell micro/nanoparticles for programmable dual drug release by emulsion electrospraying. J. Nanopart. Res..

[92] Roh K.H., Martin D.C., Lahann J. (2005). Biphasic Janus particles with nanoscale anisotropy. Nat. Mater..

[93] Roh K.H., Martin D.C., Lahann J. (2006). Triphasic nanocolloids. J. Am. Chem. Soc..

[94] Roh K.H., Yoshida M., Lahann J. (2007). Water-stable biphasic nanocolloids with potential use as anisotropic imaging probes. Langmuir.

[95] Bhaskar S., Roh K.H., Jiang X.W., Baker G.L., Lahann J. (2008). Spatioselective modification of bicompartmental polymer particles and bers via huisgen 1, 3-dipolar cycloaddition. Macromol. Rapid Comm..

[96] Bhaskar S., Pollock K.M., Yoshida M., Lahann J. (2010). Towards designer microparticles: Simultaneous control of anisotropy, shape, and size. Small.

[97] Hwang S., Roh K., Lim D.W., Wang G., Uher C., Lahann J. (2010). Anisotropic hybrid particles based on electrohydrodynamic co-jetting of nanoparticle suspensions. Phys. Chem..

[98] Lim D.W., Hwang S., Uzun O., Stellacci F., Lahann J. (2010). Compartmentalization of gold nanocrystals in polymer microparticles using electrohydrodynamic co-jetting. Macromol Rapid Commun..

[99] Yoshida M., Roh K.H., Mandal S., Bhaskar S., Lim D., Nandivada H., Deng X., Lahann J. (2009). Structurally controlled bio-hybrid materials based on unidirectional association of anisotropic microparticles with human endothelial cells. Adv. Mater..

[100] Rahmani S., Villa C.H., Dishman A.F., Grabowski M.E., Pan D.C., Durmaz H., Misra A.C., Colon-Melendez L., Solomon M.J., Muzykantov V.R., Lahann J. (2015). Long-circulating Janus nanoparticles made by electrohydrodynamic Co-jetting for systemic drug delivery applications. J. Drug Target.

[101] George M.C., Braun P.V. (2009). Multicompartmental materials by electrohydrodynamic cojetting. Angew Chem. Int. Ed. Engl..

[102] Lahann J. (2011). Recent progress in nano-biotechnology: Compartmentalized micro- and nanoparticles via electrohydrodynamic Co-jetting. Small.

